# Cross-sectional analysis of delays in care and interactions with healthcare providers among transgender and gender diverse individuals

**DOI:** 10.3389/frhs.2025.1685984

**Published:** 2025-11-17

**Authors:** Kasey A. Hill, Olveen Carrasquillo, Diana T. Medina Laabes, Vivian Colón-López

**Affiliations:** 1 Department of Neurology, Mayo Clinic, Rochester, MN, United States; 2 Department of Medicine, Miller School of Medicine, University of Miami, Miami, FL, United States; 3 University of Puerto Rico Comprehensive Cancer Center, Cancer Control and Population Sciences Division, San Juan, Puerto Rico

**Keywords:** gender diverse, transgender, delays in care, healthcare provider interactions, barriers to care

## Abstract

**Introduction:**

Timely access to positive, culturally competent healthcare experiences may be critical for transgender and gender diverse (TGD) individuals. However, gaps remain in our understanding of TGD individuals’ access to these experiences. Our aim was to determine whether TGD individuals’ likelihood of reporting delays in care and positive healthcare provider interactions differs from that of cisgender people.

**Methods:**

We analyzed survey data from 89,133 participants who enrolled in the National Institutes of Health's All of Us Research Program from 5/6/2018 to 4/1/2021. Unadjusted and adjusted logistic regressions were performed to assess the association of gender with delays in care in the past 12 months and provider interactions.

**Results:**

After adjustment, TGD individuals were more likely than cisgender men to report eight of nine reasons for care delays and more likely than cisgender women to report two of nine reasons. TGD individuals were more likely than cisgender men (OR: 2.20, 95% CI: 1.88–2.58, *p* < .001) or women (OR: 1.45, 95% CI: 1.24–1.70) to report delaying care for any reason enquired about on the survey. TGD individuals were less likely than cisgender men to report all three types of positive healthcare provider interactions and were less likely than cisgender women to report two of three types of positive interactions.

**Conclusion:**

Our findings indicate that TGD individuals may be more likely than cisgender people to experience delays in care and less likely to experience positive healthcare provider interactions. This suggests a critical need to increase TGD individuals’ access to supportive, culturally competent healthcare providers.

## Introduction

Over 1.3 million United States adults identify as transgender and gender diverse (TGD) (1). TGD individuals are people whose gender identity differs from their sex assigned at birth (2). In comparison, cisgender individuals have a gender identity that matches their sex assigned at birth (3). TGD individuals face widespread discrimination and stigma (4), which contributes to unique and urgent health challenges including high rates of mental health conditions (4), victimization via physical violence (4), substance use (5), HIV (4), chronic health conditions (4), and poor mental (6) and physical (4, 6) health. These health challenges and disparities will likely be aggravated by the increasingly hostile political climate toward TGD individuals in many parts of the United States (7).

Because of these challenges, access to supportive and culturally competent healthcare providers is of critical importance for TGD populations. Indeed, research shows supportive interactions with healthcare providers are associated with positive outcomes in TGD individuals, including better psychological wellbeing (8).

Unfortunately, TGD individuals may be unable to access healthcare because of barriers such as high rates of poverty, lack of insurance, and TGD-specific insurance exclusions (4). Even if TGD individuals can access care, these interactions may be deleterious rather than helpful. Research shows TGD individuals have negative healthcare experiences including misgendering, discrimination, verbal violence, and outright refusal to provide services (4, 8–10). It is therefore unsurprising that, in one study, almost 30% of TGD individuals reported delaying or forgoing care due to discrimination (11).

Despite previous research, gaps remain in our understanding of TGD individuals' access to positive, culturally competent healthcare interactions. To our knowledge, no current research exists comparing TGD individuals' likelihood of experiencing delays in care and positive healthcare provider interactions with that of their cisgender peers. Additionally, little is known about whether TGD people delay care for reasons other than discrimination. If so, it is unknown what those reasons are, what their prevalence is among TGD populations, and whether the prevalence differs from that of cisgender people. One possible avenue to fill these research gaps is the All of Us Research Program (AoURP).

Previous research using AoURP data demonstrated disparities in care delays by race, ethnicity, gender, and the intersection between these identities (12). This study demonstrated that it is possible to use AoURP data to study how care delays differ between different groups. However, the study excluded TGD participants. Additionally, the researchers did not examine participant interactions with healthcare providers. To address these gaps, we used data from the AoURP to examine TGD and cisgender participants’ delays in care and interactions with healthcare providers. We hypothesized that TGD individuals would be more likely to report delays in care and less likely to report positive healthcare provider interactions as compared to cisgender men.

## Methods

AoURP is an ongoing, longitudinal cohort that is funded and operated by the National Institutes of Health (13, 14). The database currently includes over 370,000 participants recruited from over 380 sites (14, 15). AoURP prioritizes recruiting participants from groups historically underrepresented in research, including TGD individuals (13). Therefore, the AoURP is an excellent resource for researchers looking to study a large, diverse sample of TGD people and/or to compare TGD individuals' experiences with those of their cisgender peers.

Upon enrollment, all AoURP participants complete “The Basics” survey, which includes demographic information such as race, ethnicity, gender identity, and sexual orientation (13, 15). Participants then have the option of completing several additional surveys, including the “Healthcare Access and Utilization” survey, which includes three questions on participants' interactions with healthcare providers and nine questions on whether participants experienced delays in care in the past 12 months (13, 15). More information on AoURP's research protocol and participants is available in The “All of Us” Research Program special report (16).

**Figure 1:**
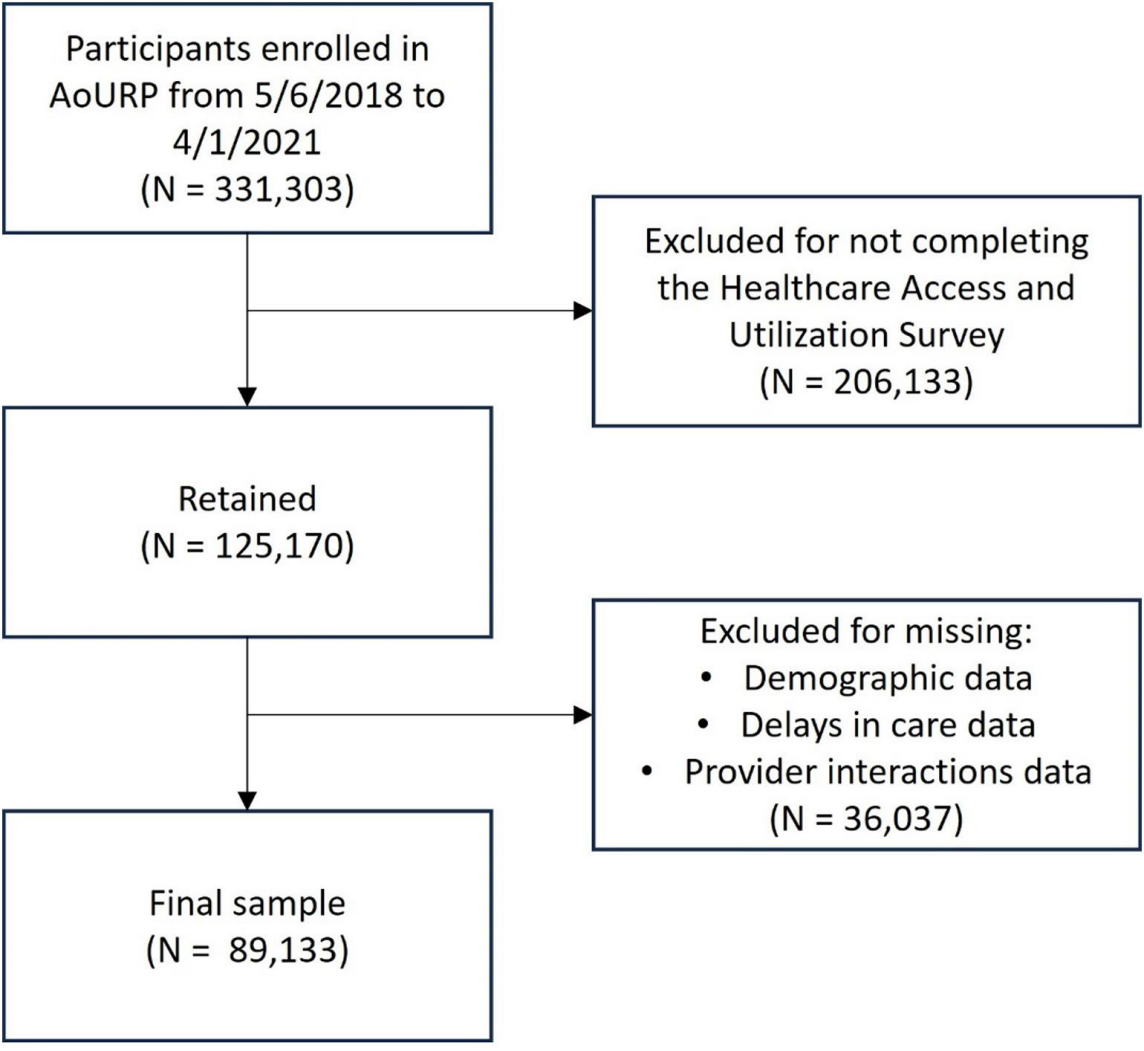
Consort diagram of inclusion/exclusion criteria and number of participants who met each criterion.

Data used in this study were collected from May 6, 2018, to April 1, 2021. During this period, 331,303 participants were enrolled in AoURP. The initial sample for this study included 125,170 adults (≥18 years) who completed AoURP's Healthcare Access and Utilization survey. Of these, 36,037 (28.8%) participants were excluded because they were missing demographic information used in the study and/or did not complete or responded “Don’t Know” to any of the questions on healthcare provider interactions or delays in care (Figure 1).

All participants who enroll in AoURP complete electronic consent modules that include explanatory videos, images, and brief text (16). AoURP participants do not undergo a separate consent process for individual research studies completed using AoURP data. In order to confirm that no separate consent process was required for our individual research study, we submitted our study to the Comprehensive Cancer Center-University of Puerto Rico Institutional Review Board. The Comprehensive Cancer Center-University of Puerto Rico Institutional Review Board deemed our study exempt because it used de-identified data from a pre-existing dataset. Therefore, the study was not considered human subjects research. AoURP requires researchers to complete yearly ethics trainings on human subjects research and AoURP data use (16). These trainings were completed by KAH, who was the only investigator with access to the AoURP data used in the study.

### Exposure

The exposure variable for all analyses was gender identity. The AoURP's “The Basics” survey provides the following gender options: Man, Woman, Non-binary, Transgender, Trans man/Transgender Man/FTM, Trans woman/Transgender Woman/MTF, Genderqueer, Genderfluid, Gender variant, Two-spirit, and Questioning or unsure of your gender identity. The Basics survey also provides the following options for sex assigned at birth: Female, Male, Intersex.

Participants were categorized as cisgender men if they self-reported their gender as “man” and their sex assigned at birth as “male.” Participants were categorized as cisgender women if they reported their gender as “woman” and their sex assigned at birth as “female.” All other sex/gender combinations were categorized as TGD. The decision was made to create a single TGD category because many of the gender/sex assigned at birth combinations were endorsed by a small number of participants. Therefore, reporting more granular gender/sex assigned at birth combinations would have put participants at risk of de-identification and would have limited the analyses' statistical power.

### Outcomes

Outcome variables were participant responses to the delays in care and healthcare provider interaction items from the Healthcare Access and Utilization survey. Delays in care items use dichotomous yes/no response choices. In addition to delays in care items included on the survey, a dichotomous “delays in care for any reason” variable was created by grouping all participants who responded “yes” to one or more delays in care item vs. all other participants.

Healthcare provider interaction response choices use a 4-point scale (always, most of the time, some of the time, none of the time). Based on the frequency distribution of responses, response choices were combined to create a dichotomous “all or most of the time” vs. “some or none of the time” variable.

### Statistical analysis

Descriptive statistics were calculated for demographic, delays in care, and healthcare provider interaction variables. Logistic regression was used to calculate odds ratios (OR) for the association between gender identity, delays in care, and provider interactions. Analyses were performed in two ways: unadjusted and adjusted for race/ethnicity, sexual orientation, employment status, education, income, marital status, health insurance status, and age (Table 1). All adjustment variables were identified using items from AoURP's The Basics survey. Adjustment variables were chosen based on preexisting literature (17–22) and maintained in the model following collinearity and likelihood-ratio testing. All logistic regressions were performed twice, using two different reference groups: cisgender men and cisgender women. Significance was set at *p* < .05.

**Table 1:** Sample demographics by gender identity.

Demographic	Cisgender man *N* = 29,278 (32.8%)	Cisgender woman *N* = 58,880 (66.1%)	Transgender and gender minority *N* = 975 (1.1%)	*P* value^a^
Race/ethnicity				<.001
White	23,438 (80.1%)	44,004 (74.7%)	725 (74.4%)	
Asian	1,109 (3.8%)	1,790 (3.0%)	37 (3.8%)	
Black	1,646 (5.6%)	5,289 (9.0%)	40 (4.1%)	
Hispanic	1,593 (5.4%)	4,560 (7.7%)	58 (5.9%)	
Other or multiple races	1,492 (5.1%)	3,237 (5.5%)	115 (11.8%)	
Sexual Orientation				<.001
Straight	25,913 (88.5%)	54,001 (91.7%)	136 (13.9%)	
Lesbian, gay, or bisexual	3,128 (10.7%)	3,985 (6.8%)	443 (45.4%)	
Other or multiple sexual orientations	237 (0.8%)	894 (1.5%)	396 (40.6%)	
Employment status				<.001
Employed	13,905 (47.5%)	30,904 (52.5%)	447 (45.8%)	
Not employed (including retired)	11,966 (40.9%)	20,371 (34.6%)	220 (22.6%)	
Student	656 (2.2%)	1,563 (2.7%)	63 (6.5%)	
Multiple employment options	2,751 (9.4%)	6,042 (10.3%)	245 (25.1%)	
Education				<.001
Some college or more	26,665 (91.1%)	52,383 (89.0%)	864 (88.6%)	
High school or less	2,613 (8.9%)	6,497 (11.0%)	111 (11.4%)	
Income				<.001
Not low income	24,481 (83.6%)	46,348 (78.7%)	585 (60.0%)	
Low income	4,797 (16.4%)	12,532 (21.3%)	390 (40.0%)	
Marital Status				<.001
Married or living with partner	20,336 (69.5%)	34,232 (58.1%)	399 (40.9%)	
Divorced, widowed, or separated	3,736 (12.8%)	12,428 (21.1%)	119 (12.2%)	
Never married	5,206 (17.8%)	12,220 (20.8%)	457 (46.9%)	
Health insurance status				0.26
Insured	28,674 (97.9%)	57,565 (97.8%)	953 (97.7%)	
Not insured	604 (2.1%)	1,315 (2.2%)	22 (2.3%)	
Age				<.001
Mean (standard deviation)	59.6 (16.4)	53.8 (16.3)	39.6 (14.3)	

a *P* values were obtained using Pearson *χ*² Test or one-way Analysis of Variance (ANOVA), as appropriate. Significance was set at *p* < .05.

Data was accessed and analyses were performed using R environment with R version 4.2.2 Patched (2022-11-10 r83330) in AoURP Researcher Workbench, which is a secured, cloud-based platform used by AoURP to make data accessible to approved researchers (13, 15). The dataset supporting the conclusions of this article is available on AoURP Researcher Workbench (15).

## Results

There were 331,303 AoURP participants enrolled from 5/6/2018 to 4/1/2021, of whom 206,133 did not complete the Healthcare Access and Utilization survey (Figure 1) and were excluded from the final sample. Of excluded participants, 200,225 had sex assigned at birth of “male” or “female” and provided their gender identity. Among these excluded participants, 81,977 (40.9%) were cisgender men, 116,910 (58.4%) were cisgender men, and 1338 (0.7%) were TGD.

The final sample of 89,133 was 32.8% cisgender men, 66.1% cisgender women, and 1.1% TGD individuals (Table 1). Participants' mean age was 55.6 (standard deviation 16.6), 23.5% reported non-White race/ethnicity, and 97.8% had health insurance.

Of all participants, 37.9% reported experiencing a delay in care in the last 12 months for any reason. The most commonly reported reasons for experiencing a delay in care were “had to pay out of pocket for some or all of the procedure” (18.1%), “nervous about seeing a healthcare provider” (13.5%), and “couldn’t get time off work” (11.8%). The majority of participants reported their healthcare provider always or most of the time “treated you with respect” (96.8%), “gave you information that was easy to understand” (93.8%), and “asked your opinions or beliefs” (56.7%).

### Delays in care

Before adjustment, TGD participants were more likely than cisgender men to report delaying care in the past 12 months for all nine reasons enquired about on the survey (Table 2). TGD participatns were more likely than cisgender women to report delaying care for seven of nine reasons. TGD individuals most commonly reported delaying care because they were “nervous about seeing a healthcare provider” (47.7%), “had to pay out of pocket” (33.2%), and “couldn’t get time off work” (24.9%). A higher percentage of TGD individuals than of cisgender men or women reported delaying care for any reason (TGD individuals: 71.6%, cisgender men: 28.0%, cisgender women: 42.2%, *p* < *.*001 for both comparisons).

**Table 2:** Results of unadjusted and adjusted logistic regressions for delays in care by gender.

In the past 12 months, delayed care because… (Response = “Yes”)	Cisgender Men *N* = 29,278 (32.8%) Number (%)	Cisgender Women *N* = 58,880 (66.1%) Number (%)	Gender Minority *N* = 975 (1.1%)				
			Number (%)	Comparison group = cisgender men		Comparison grou*p* = cisgender women	
				Unadjusted odds ratio (95% confidence interval), *p* value	Adjusted^a^ odds ratio (95% confidence interval), *p* value	Unadjusted odds ratio (95% confidence interval), *p* value	Adjusted^a^ odds ratio (95% confidence interval), *p* value
Didn’t have transportation	1,351 (4.6%)	3,997 (6.8%)	197 (20.2%)	5.23 (4.43–6.16), *p* < .001	1.70 (1.40–2.06), *p* < .001	3.47 (3.00–4.06), *p* < .001	1.44 (1.19–1.74), *p* < .001
Live in a rural area where distance to the healthcare provider is too far	578 (2.0%)	1,801 (3.1%)	63 (6.5%)	3.43 (2.60–4.45), *p* < .001	1.55 (1.14–2.07), *p* = .004	2.19 (1.67–2.81) *p* < .001	1.19 (0.88–1.59), *p* = .23
Nervous about seeing a healthcare provider	2,527 (8.6%)	9,040 (15.4%)	465 (47.7%)	9.65 (8.46–11.01), *p* < .001	3.03 (2.61–3.51), *p* < .001	5.04 (4.43–5.72), *p* < .001	1.90 (1.65–2.20), *p* < .001
Couldn’t get time off work	2,244 (7.7%)	8,042 (13.7%)	243 (24.9%)	4.00 (3.43–4.65), *p* < .001	1.60 (1.34–1.90), *p* < .001	2.10 (1.81–2.42), *p* < .001	1.08 (0.91–1.27), *p* = .39
Couldn’t get childcare	309 (1.1%)	2,173 (3.7%)	27 (2.8%)	2.67 (1.75–3.90), *p* < .001	1.08 (0.69–1.63), *p* = .73	0.74 (0.49–1.07), *p* = .13	0.47 (0.30–0.70), *p* < .001
Provide care to an adult and could not leave him/her	279 (1.0%)	1,186 (2.0%)	27 (2.8%)	2.96 (1.94–4.33), *p* < .001	2.72 (1.72–4.14), *p* < .001	1.38 (0.92–1.99), *p* = .09	1.33 (0.85–1.99), *p* = .19
Couldn’t afford the copay	1,653 (5.6%)	6,005 (10.2%)	217 (22.3%)	4.78 (4.07–5.60), *p* < .001	1.82 (1.52–2.18), *p* < .001	2.52 (2.15–2.93), *p* < .001	1.21 (1.01–1.43), *p* = .03
Deductible was too high/or could not afford the deductible	2,165 (7.4%)	7,108 (12.1%)	178 (18.3%)	2.80 (2.36–3.30), *p* < .001	1.38 (1.14–1.66), *p* < .001	1.62 (1.37–1.91), *p* < .001	0.98 (0.82–1.17), *p* = .82
Had to pay out of pocket for some or all of the procedure	4,023 (13.7%)	11,784 (20.0%)	324 (33.2%)	3.12 (2.72–3.58), *p* < .001	1.57 (1.35–1.82), *p* < .001	2.0 (1.74–2.28), *p* < .001	1.16 (0.99–1.34), *p* = .06
For any reason	8,199 (28.0%)	24,864 (42.2%)	698 (71.6%)	6.48 (5.63–7.47), *p* < .001	2.20 (1.88–2.58), *p* < .001	3.45 (3.01–4.00), *p* < .001	1.45 (1.24–1.70), *p* < .001

a Adjusted logistic regression controlled for race/ethnicity, sexual orientation, employment status, education, income, marital status, health insurance status, and age. Significance was set at *p* < .05.

After adjusting for all variables, TGD participants were more likely than cisgender men to report delaying care for every reason except “couldn’t get childcare” [OR: 1.08, 95% confidence interval (CI) 0.69–1.63]. In particular, TGD individuals were more likely to report delaying care because they were nervous about seeing a healthcare provider (OR: 3.03, 95% CI: 2.61–3.51), provided care to an adult and could not leave him/her (OR: 2.72, 95% CI: 1.72–4.14), and couldn't afford the copay (OR: 1.82, 95% CI: 1.52–2.1]). TGD individuals were more likely than cisgender men to report delaying care for any reason (OR: 2.20, 95% CI: 1.88–2.58).

After adjusting for all variables, TGD participants were more likely than cisgender women to report delaying care because they didn't have access to transportation (OR: 1.44, 95% CI: 1.19–1.74) and because they were nervous about seeing a healthcare provider (OR: 1.90, 95% CI: 1.65–2.20). TGD participants were less likely than cisgender women to report delaying care because they couldn't get childcare (OR: 0.74, 95% CI: 0.30–0.70). TGD individuals were more likely than cisgender women to report delaying care for any reason (OR: 1.45, 95% CI: 1.24–1.70).

### Healthcare provider interactions

Before adjustment, TGD participants were less likely (*p* < .001 for all comparisons) than cisgender men or women to report their healthcare provider always or most of the time treated them with respect (TGD individuals: 89.5%, cisgender men: 97.3%, cisgender women: 96.6%), gave information that was easy to understand (TGD individuals: 87.5%, cisgender men: 95.0%, cisgender women: 93.4%), and asked their opinions or beliefs (TGD individuals: 47.8%, cisgender men: 58.9%, cisgender women: 55.8%; Table 3).

**Table 3:** Results of unadjusted and adjusted logistic regressions for healthcare provider interaction by gender.

How often did your doctors or healthcare providers… (Response = “Always” or “Most of the time”)	Cisgender men *N* = 29,278 (32.8%) Number (%)	Cisgender women *N* = 58,880 (66.1%) Number (%)	Gender minority *N* = 975 (1.1%)				
			Number (%)	Comparison grou*p* = cisgender men		Comparison group = cisgender women	
				Unadjusted odds ratio (95% confidence interval), *p* value	Adjusted^a^ odds ratio (95% confidence interval), *p* value	Unadjusted odds ratio (95% confidence interval), *p* value	Adjusted^a^ odds ratio (95% confidence interval), *p* value
Treat you with respect	28,500 (97.3%)	56,865 (96.6%)	873 (89.5%)	0.23 (0.19–0.29), *p* < .001	0.59 (0.46–0.75), *p* < .001	0.30 (0.25–0.38), *p* < .001	0.62 (0.49–0.79), *p* < .001
Tell or give you information about your health and healthcare that was easy to understand	27,823 (95.0%)	54,971 (93.4%)	853 (87.5%)	0.37 (0.30–0.45), *p* < .001	0.65 (0.53–0.81), *p* < .001	0.50 (0.41–0.61), *p* < .001	0.75 (0.61–0.94), *p* = .01
Ask for your opinions or beliefs about your medical care or treatment	17,245 (58.9%)	32,834 (55.8%)	466 (47.8%)	0.64 (0.56–0.73), *p* < .001	0.84 (0.74–0.97), *p* = .02	0.73 (0.64–0.83), *p* < .001	0.92 (0.81–1.06), *p* = .27

a Adjusted logistic regression controlled for race/ethnicity, sexual orientation, employment status, education, income, marital status, health insurance status, and age. Significance was set at *p* < .05.

After adjusting for all variables, TGD participants were still less likely than cisgender men to report all three types of positive provider interactions: treated you with respect (OR: 0.59, 95% CI: 0.46–0.75), gave you information that was easy to understand (OR: 0.65, 95% CI: 0.53–0.81), and asked about your opinions or beliefs (OR: 0.84, 95% CI: 0.74–0.97). TGD participants were less likely than cisgender women to report that their healthcare provider treated them with respect (OR: 0.62, 95% CI: 0.49–0.79) and gave them information that was easy to understand (OR: 0.75, 95% CI: 0.61–0.94).

## Discussion

Using data from a large national sample, we found that TGD individuals were more likely than cisgender men to report eight of nine reasons for experiencing delays in care and less likely to report all three types of positive interactions with healthcare providers. TGD individuals were more likely than cisgender women to report two of nine reasons for experiencing delays in care and were less likely to report two of three types of positive interactions with healthcare providers. Additionally, TGD participants were more likely than cisgender men or women to report delaying care for any reason.

The inequities in care delays and provider interactions seen in this study may contribute to the disparities in mental and physical health faced by TGD individuals (6). Additionally, clinical encounters may represent a missed opportunity to address TGD populations' unique health challenges, including high rates of physical violence and suicidality (4, 8).

Strikingly, TGD participants had three times greater odds relative to cisgender men of delaying care because they were nervous to see a healthcare provider. TGD individuals may be nervous to see providers for many reasons, such as fear of receiving a difficult diagnosis. However, one major contributor may be fear of experiencing discrimination, which previous research shows is a reason for which TGD individuals commonly report delaying care (11). One survey of TGD Americans found that thirty percent of participants delayed or did not seek needed health care due to discrimination (11). This result is consistent with the 47.7% of TGD participants in our study who reported delaying care because they were nervous about seeing a healthcare provider.

TGD individuals also frequently reported delaying care for financial and vocational reasons, such as having to pay out of pocket or being unable to get time off work. TGD individuals may be more likely than cisgender people to experience financial or work-based delays in care secondary to high rates of poverty (4, 6) and low rates of health insurance (4). However, this study found disparities persisted between cisgender men and TGD individuals after controlling for income and insurance status. Additionally, TGD individuals in this cohort reported a high rate of health insurance. The reasons for this discrepancy are likely multifactorial. Contributors may include TGD-specific exclusions in healthcare coverage (4) and higher rates of public insurance among TGD people than in the general population (23).

Even if TGD individuals are able to access care, provider interactions may be of lower quality than those experienced by cisgender individuals. Our study found TGD individuals are less likely than cisgender people to report their healthcare providers treat them with respect, give information that is easy to understand, and ask for their opinions or beliefs. These results are consistent with previous research in which TGD individuals reported their providers are unknowledgeable about TGD health, misgender them, are openly transphobic, and sometimes refuse to treat them (10, 24).

### Limitations

One of this study's major limitations is that it does not differentiate between TGD participants who identify as men, women, and nonbinary/genderqueer. These groups differ in meaningful ways (6) and likely have diverse experiences with the healthcare system (11). Our study also did not explore whether the intersection between gender and other identities, such as race/ethnicity and income, impacted delays in care and provider interactions. More granular analyses of gender identity and intersectional analyses were not performed due to concerns for sample size and participant anonymity. As AoURP participation increases, future research can more fully explore these subjects.

AoURP was not designed with a formal statistical sampling method, which may limit the generalizability of results. For example, TGD participants in this study reported a high rate of health insurance coverage, despite the fact that TGD people in the United States often lack access to health insurance (4). Despite this limitation, the AoUPRP cohort has been validated by replicating the findings of earlier studies on diabetes, depression medications, and the relationship between smoking and cancer (13). Additionally, this study likely would not have been possible without the AoURP's intentional over-recruitment of TGD individuals; the prevalence of TGD study participants was more than twice that estimated for the overall United States adult population (1).

Many AoURP participants (62.2%) did not complete the “Healthcare Access and Utilization” survey and were excluded from the final sample. There may be meaningful differences between participants who did and did not complete the survey. For example, 0.7% of excluded participants were TGD, as compared to 1.1% of participants included in the final sample. This suggests that TGD individuals may have been more likely than their cisgender counterparts to complete the Healthcare Access and Utilization survey, potentially biasing the results.

AoURP did not collect data on the type of medical care that participants were attempting to access (primary care, specialty care, etc.) The reasons that for delays in care may differ between different types of care. AoURP also did not collect data on whether participants delayed care due to the COVID-19 pandemic. It is possible that the pandemic caused an increase in care delays for reasons that are enquired about on AoURP surveys. However, previous research using the AoURP dataset found that a similar percentage of participants reported delaying care both before and after the pandemic (12).

A final limitation is that TGD participants may have experienced delays in care for reasons not accounted for by this study. For example, electronic health records may not accurately reflect patients' chosen names and gender or may restrict access to certain exams, such as cervical Papanicolaou, based on gender (25). Although AoURP offers a free response option for participants to report other reasons for delays in care, information on the percentage of participants who chose the free response option and their precise answers is not currently available.

### Implications

Multilevel efforts should be implemented to address the disparities seen in this study. On an individual level, physicians should educate themselves about TGD health and work to identify and address their own biases. At the system/organizational level, hospitals should identify and eliminate barriers that prevent TGD individuals from accessing care. Medical schools and hospitals could implement training for students and staff on TGD patients.

From a structural/policy perspective, governmental and professional organizations should take steps to ensure TGD individuals have equitable access to respectful and competent healthcare providers. Steps could include increasing access to health insurance and eliminating economic barriers to accessing care, developing outreach programs, and setting national standards for TGD patient care. Unfortunately, many governmental bodies in the United States have taken the opposite approach. In recent years, multiple states have passed laws and policies designed to limit TGD individuals' access to healthcare and to increase stigma against TDG people (7). These laws may worsen TGD individuals' likelihood of experiencing delays in care, access to culturally competent healthcare providers, and other health disparities (7). To improve the health and wellbeing of TGD people, reversing anti-TGD laws and policies must be a critical priority.

## Data Availability

Publicly available datasets were analyzed in this study. This data can be found here: AoURP Researcher Workbench.
